# Analysis of Multiple Mycotoxins in the Qatari Population and Their Relation to Markers of Oxidative Stress

**DOI:** 10.3390/toxins13040267

**Published:** 2021-04-08

**Authors:** Belqes Al-Jaal, Aishah Latiff, Sofia Salama, Huda Mohamed Hussain, Noora Abdulaziz Al-Thani, Noor Al-Naimi, Noof Al-Qasmi, Peter Horvatovich, Morana Jaganjac

**Affiliations:** 1 Anti-Doping Lab Qatar, Sport City Road, Doha P.O. Box 27775, Qatar; baljaal@adlqatar.qa (B.A.-J.); ssalama@adlqatar.qa (S.S.); hhussain@adlqatar.qa (H.M.H.); noalthani@adlqatar.qa (N.A.A.-T.); nalnaimi@adlqatar.qa (N.A.-N.); nalqassmi@adlqatar.qa (N.A.-Q.); 2 School of Pharmaceutical Sciences, University of Science Malaysia, Gelugor 11700, Pulau Pinang, Malaysia; alselasih1@gmail.com; 3 Department of Analytical Biochemistry, Groningen Research Institute of Pharmacy, University of Groningen, 9713 AV Groningen, The Netherlands; P.L.Horvatovich@rug.nl; 4 Division of Molecular Medicine, Rudjer Boskovic Institute, Bijenicka 54, 10000 Zagreb, Croatia

**Keywords:** mycotoxin exposure, human biomonitoring, oxidative stress

## Abstract

Mycotoxins are naturally occurring food toxins worldwide that can cause serious health effects. The measurement of mycotoxin biomarkers in biological fluids is needed to assess individuals’ exposure. The aim of this study was to investigate the incidence of mycotoxins in the Qatari population. Serum samples from 412 adults and urinary samples from 559 adults were analyzed for the presence of mycotoxin biomarkers. Multimycotoxin approaches have been applied, using liquid chromatography mass spectrometry methods. Samples were further analyzed for the oxidative stress markers and compared with regard to the incidence of mycotoxins. The presence of mycotoxins was identified in 37% of serum samples and in less than 20% of urine samples. It was found that 88% of positive of the samples were positive for only one mycotoxin, while 12% of positive samples had two or more mycotoxins. Trichothecenes and zearalenone metabolites were most commonly detected mycotoxins, followed by aflatoxins, roquefortine C and mycophenolic acid. The presence of mycotoxins was found to positively correlate with oxidative stress markers. The obtained results illustrate the importance of mycotoxin biomonitoring studies in humans and the need to elucidate the underlying mechanisms of mycotoxin-induced toxicity.

## 1. Introduction

Mycotoxins are common food toxins produced by fungi of adverse agricultural crops worldwide [1]. Despite the set regulatory limits and continuous monitoring of food and feed, exposure to mycotoxins remains a global issue. The ingestion of mycotoxins is the most common route of exposure, although exposure can occur via the dermal and respiratory routs as well. Depending on the type of mycotoxin exposure characterised by the concentration and duration of exposure, acute (short exposure at high concentration) and chronic (long exposure at low and medium concentration) mycotoxicosis might be developed. Mycotoxins have been implicated in the pathology of various diseases, among which is cancer [2,3]. Based on the epidemiological data and experimental studies, most aflatoxins (AFs) have been classified according to the International Agency of Research on Cancer (IARC) as group 1 carcinogens to humans, while fumonisins (FBs), ochratoxin A (OTA) and sterigmatocystin (STE) are classified as possibly carcinogenic to humans, group 2B [4]. Although the underlying mechanisms of toxicity for some mycotoxins are well known, for others they still remain to be elucidated. In addition, coexposure to different mycotoxins can have synergistic effects and impair health even at low concentrations. Thus, the continuous monitoring and risk assessment of human mycotoxin exposure is crucial. Recently, we have reported the first mycotoxin biomonitoring in Qatar [5]. Although the study included a small number of subjects and looked only into presence of mycotoxins in serum samples, it was evident that more than 35% of residents are at risk of mycotoxin exposure. Furthermore, food analyses in Qatar and across the region, showed that certain types of food are frequently contaminated with mycotoxins even above the allowed regulatory limits [3,6]. In addition, the overproduction of reactive oxygen species (ROS) and altered redox homeostasis have been implicated in the mechanism of toxicology of a number of mycotoxins [7]. Excessive ROS, based on their reactivity and the proximity of target molecules, can modulate signaling pathways, modify macromolecules or induce cell death [8,9,10]. The peroxidation of lipids leads to the formation of reactive aldehydes that have a longer lifetime than ROS, can move across membranes and affect targets distant from the initial site of oxidative injury [11,12]. Thus, in the current study, the aim was to provide an epidemiological “snapshot” of population exposure to mycotoxins by assessing the incidence of their biomarkers in the serum and urine samples of larger cohort using multimycotoxin approaches. In order to understand the impact of mycotoxin exposure on body redox homeostasis, oxidative stress markers in serum and urine have been correlated with the presence of mycotoxins.

## 2. Results

Serum and urine samples of residents of Qatar were analyzed for the presence of multiple mycotoxins and markers of oxidative stress. The characteristics of both cohorts are shown in Table 1.

Among those who declared their overall health status, more than 80% of all subjects were of excellent or good health and almost 65% did not smoke, i.e., were either non-smokers or former smokers. The incidence of mycotoxins in serum samples of Qatari residents is shown in Table 2. The concentration range for those mycotoxins detected in samples is included as well. We found that 63% of the serum samples were negative for the presence of mycotoxins. Aflatoxin B1 (AFB1) and aflatoxin M1 (AFM1) were not detected in a single sample while aflatoxin B2 (AFB2) and aflatoxin G2 (AFG2) were detected in 0.2% and 6.3% of the samples, respectively. Moreover, although deoxynivalenol (DON) was not detected, its two acetylated analogs, 15-AcDON and 3-AcDON were found in 0.2% and 6.3% of the samples. Besides 3-AcDON, mycophenolic acid (MPA) and neosolaniol (NEO) were the most commonly measured mycotoxins in serum samples. On the contrary, AFB1, AFM1, DON, fusarenone X (FusX), HT-2 toxin (HT-2), roquefortine C (ROC), α-Zearalenol (a-ZEL) and β-Zearalenol (b-ZEL) were not detected in serum samples analyzed. The mycotoxins with an incidence of below 5% were OTA, Ochratoxin B (OTB), Cyclopiazonic acid (CPA), STE and zearalenone (ZEN).

The results of the multimycotoxin analyses of the 14 mycotoxins found in the urine samples are shown on Table 3. Compared to the serum samples, the incidence of mycotoxins was lower in the urine samples, with 80% of the samples found to be negative.

The highest incidences, 3.9 % and 7.5%, were found for a-ZEL and b-ZEL, respectively. ROC is another mycotoxin with high incidence in urine samples (4.3%). The mycotoxins with incidence below 3% are AFB1, AFB2, AFG2, AFM1, CIT, FB1, OTB, STE and T-2, while CPA and OTA were not detected in a single sample.

**Figure 1:**
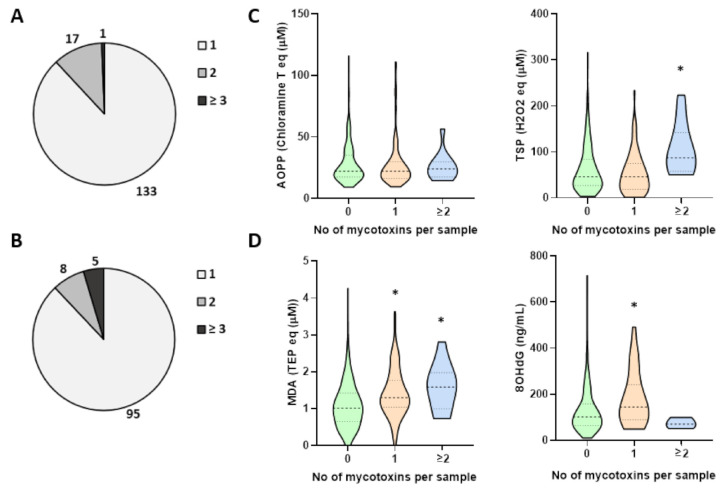
Incidence of multiple mycotoxins in urine and serum samples and their correlation with parameters of oxidative stress. (**A**) Serum and (**B**) urine samples with 1, 2 or ≥3 mycotoxins per sample. Oxidative stress parameters measured in (**C**) serum as advanced oxidation protein products (AOPP) and total serum peroxides (TSP) and in (**D**) urine as malondialdehyde (MDA) and 8-hydroxydeoxyguanine (8OHdG). Dash lines in violin plots represent the median while the upper and lower dotted lines correspond to 1st and 3rd quartile, * significance *p* < 0.05 compared to control.

Further analysis of the potential impact of mycotoxins on body redox homeostasis is shown in Figure 1. From 151 serum samples positive for mycotoxins, almost 90% of them contained only one mycotoxin, while 12% samples contained two or more mycotoxins (Figure 1A). Similarly, of 108 positive urine samples, most (88%) were positive for only one mycotoxin, while 12% had two or more mycotoxins (Figure 1B). These findings were used for further grouping of samples according to those negative for the presence of mycotoxins, the group containing only one mycotoxin per sample and the group with multiple mycotoxins.

The total serum peroxides (TSP) and advanced oxidation protein products (AOPP) were the markers of oxidative stress analyzed in the serum (Figure 1C) while lipid peroxidation end product malondialdehyde (MDA) and 8-hydroxydeoxyguanine (8OHdG) were the parameters of oxidative stress analyzed in the urine (Figure 1D). AOPPs did not significantly differ in respect to presence of mycotoxins (*p* > 0.05) while TSP were significantly elevated in the samples containing multiple mycotoxins (*p* < 0.05). Interestingly, the urinary levels of MDA significantly increased with the presence of mycotoxins (*p* < 0.05). DNA damage marker was also elevated in the samples positive for one mycotoxin (*p* < 0.05).

Additional analysis was performed for mycotoxins identified with a higher incidence in the serum or the urine samples with oxidative stress parameters. The TSP levels were increased in the serum samples positive for MPA by 52% (*p* < 0.05) and by 39% in samples positive for AFs (*p* = 0.057). Moreover, the MDA level was found elevated by 42% in the urine samples positive for a- and/or b-ZEL (*p* < 0.05). The urine samples positive for ROC had significantly elevated both MDA and 8OHdG by 39% and 98%, respectively (*p* < 0.05 for both). For the other mycotoxin biomarkers measured in serum or urine, no significant differences in respect to oxidative stress parameters were observed.

## 3. Discussion

In the past, a large number of biomonitoring studies measured only one mycotoxin; since then multimycotoxin methods are becoming more popular [13]. The measurement of multiple-mycotoxins, instead of only the most common or the most harmful ones, is preferred as it gives better insight for the population exposure. In addition, studies have shown that coexposure to multiple mycotoxins could seriously alter health even if the mycotoxins are present at low concentration [14]. Recently, we reported a small scale biomonitoring study in Qatar, analyzing the serum samples of only 46 volunteers [5]. That was the first human biomonitoring study conducted in Qatar, and it included both Qatari and non-Qatari volunteers. The present study included a larger number of samples collected only from Qatari nationals. Among the mycotoxins analyzed, those most frequently detected in serum samples were MPA and NEO. MPA can be produced by *Penicillium* species but it is also used as an immunosuppressant medication, while NEO can be produced by *Fusarium* species and is also a metabolite of T-2 [15]. Acetylated derivatives of another trichothecene, DON, were also present with higher incidence in serum samples. The information on the food contamination with trichothecenes in Qatar and the surrounding region is limited [3]. In 2002, 11% of cereals and cereal products analyzed in Qatar contained DON [16]. A recent study reported that close to 40% of baby cereal food in Qatar was contaminated with T-2 (range from 1 to ≥10 μg/kg) while more than 90% (range from 20 to ≥200 μg/kg) was found to be positive for DON [17]. Moreover, a study in the region (Jeddah, Saudi Arabia), reported that all wheat collected from Jeddah market, regardless of its origin, was contaminated with DON (range from 15.24 to 803.22 μg/kg) [18]. These findings highlight that closer monitoring of food contamination with trichothecenes, both imported and locally grown in Qatar, is needed.

Aflatoxins were also frequently detected in both urine and serum samples. We recently reported that 6% of cereals, 7% of nuts and more than 64% of spices contained AFs [6]. Among those food samples, nuts and spices frequently exceeded the regulatory limit. This study identified close to 5% of urine and 6.6% of serum samples positive for AFs, which could be due to high intake of nuts and spices in Qatar. Furthermore, the presence of OTs was documented in 5% of the serum samples. This is in agreement with the analysis of food in Qatar, where almost 5% of the analyzed samples contained OTs, with the highest concentration reported for dried fruits, nuts and spices [6].

Interestingly, although almost all serum samples were negative for the presence of ZEN or its metabolites, close to 11% of urine samples contained ZEN metabolites. This could be due to the metabolism or higher sensitivity of the method used for the urine samples. 

Moreover, we recorded almost 5% of the urine samples as being positive for ROC. ROC is a neurotoxic metabolite of *Penicillium* species and is considered one of the most important fungal contaminants in fermented foods and beverages [19]. The ROC concentration in blue cheese can reach to 5.45 mg/kg and it also readily co-occurs with MPA [20]. Thus, dairy products in Qatar should be carefully monitored. Among the mycotoxins with lower incidence in samples from Qatari residents were CPA, STE, CIT and FB1—still, their potential negative effects should not be neglected.

Earlier in vitro studies have shown that the mechanism of mycotoxin induced toxicity of AFB1, FB1, OTA, ROC, DON and acetylated analogs of DON is mediated by excessive ROS formation [21,22,23,24,25]. ZEN and CIT also modulate redox homeostasis by altering antioxidant defenses and promoting ROS [26,27]. In the present paper we have noticed that regardless of the type of mycotoxin, samples positive for any mycotoxin had elevated urinary MDA which was further elevated with increasing number of mycotoxins per sample. In addition, the mycotoxin positive samples had elevated urinary 8OHdG and total serum peroxides. These data further confirm earlier findings that among the mechanisms of mycotoxin-induced toxicity is the overproduction of ROS, triggering damage to macromolecules, such as nucleic acids and lipids. A disturbance in redox homeostasis can lead to deregulated cellular, molecular and physiological pathways, which are activated to repair oxidative stress-induced damage and can introduce different somatic mutations in cells, resulting in either physiological or pathological adaptation. The monitoring of oxidative stress parameters could indicate the clearance of mycotoxins from the body.

## 4. Conclusions

This is the first mycotoxin biomonitoring study in Qatar on large cohort. Among the mycotoxins analyzed, the trichothecene and ZEN metabolites were the most frequently detected, while AFs, MPA and ROC had lower prevalence. The obtained results highlight the importance of the continuous monitoring of population exposure to multiple mycotoxins and provide the basis for the future risk assessment studies. Moreover, the monitoring of food contamination should be extended to other, less common, mycotoxins, to their metabolites as well as to a larger variety of food types. Finally, continuous improvement of multimycotoxin detection methods is needed to increase the variety of mycotoxins monitored and to gain better sensitivity.

## 5. Materials and Methods

### 5.1. Chemicals and Reagents

The HPLC grade acetonitrile (VWR), glacial acetic acid (Sigma-Aldrich, Darmstadt, Germany), HPLC grade formic acid (Merck, Darmstadt, Germany) and deionized water obtained from a Milli-Q Integral 5 water purification system (Millipore, MA, USA) were used for the sample preparation and LC–MS/MS analysis.

### 5.2. Reference Materials

AFB1, AFB2, AFM1 and AFG2 were purchased from Sigma-Aldrich (Germany). OTA, OTB, FB1, ^13^C_17_-AFB1 and ^13^C_20_-OTA were purchased from Romer Labs (Austria). ZEN, a-ZEL, b-ZEL, DON, 3-AcDON, 15-AcDON, NEO, HT-2, CIT, STE, T-2, ROC, MPA, CPA, Fus-X, ^13^C_22_-HT-2, ^13^C_15_-DON and ^13^C_24_-T-2 were purchased from Chiron (Trondheim, Norway).

Standard stock solutions were prepared in acetonitrile and stored at −20 °C in amber glass vials. The mixture of the working solution was prepared by diluting each stock solution in acetonitrile. A combined multi-internal standard (ISTD) working solution contained ^13^C_17_-AFB1 (2 ng/mL), ^13^C_20_-OTA (20 ng/mL), ^13^C_22_-HT-2 (30 ng/mL), ^13^C_15_-DON (50 ng/mL) and ^13^C_24_-T-2 (10 ng/mL).

All other chemicals and standards were obtained from Sigma-Aldrich (Germany) unless specified otherwise.

### 5.3. Study Population and Biological Samples

Serum (*n* = 421) and urine (*n* = 559) samples, together with the questionnaire data to obtain information regarding age, weight, food habits and general health were obtained from Qatar Biobank, Doha Qatar (Research Application No.: QF-QBB-RES-ACC-0109, IRB Protocol: Ex-2018-RES-ACC-0109-0051).

### 5.4. Sample Preparation

Serum samples were prepared as described before [5]. Briefly, serum samples were spiked with ISTD working solution and incubated at room temperature for 30 min, followed by protein precipitation with acetonitrile/water/acetic acid (80/19/1, *v*/*v*/*v*) for 30 min. Proteins were pelleted by centrifugation at 8517 g for 15 min at 4 °C and 500 μL of supernatant transferred into a new vial, evaporated to dryness at 40 °C with a stream of nitrogen and reconstituted in 50 µL acetonitrile/water (15/95, *v*/*v*).

Urine samples (100 µL) were spiked with 20 µL of ISTD mixture, followed by addition of 25 µL of β-glucuronidase from *Escherichia coli* (Sigma-Aldrich, Darmstadt, Germany) and incubation for 24 h at 37 °C. Samples were then diluted to 500 µL with ACN/water (20/80, *v*/*v*) containing 0.1% FA and filtered through 0.45 µm PVDF filter (Merck Millipore) directly into LC vial.

### 5.5. MultiMycotoxin Analysis of Serum Samples by UHPLC–MS/MS

The mycotoxin biomarkers analyzed in serum samples were 15-AcDON, 3-AcDON, AFB1, AFB2, AFG2, AFM1, CPA, DON, FusX, HT-2, MPA, NEO, OTA, OTB, ROC, STE, ZEN, a-ZEL, b-ZEL. 

The multimycotoxin MRM UPLC–MS/MS analyses of serum samples was performed on a Waters Xevo TQ-S triple quadrupole mass spectrometer (Waters Corp, Wilmslow, UK) coupled with Waters Acquity UPLC and controlled by Masslynx software (version 4.1). The method used vas fully validated according to the National Association of Testing Authorities (NATA) guidelines for the validation and verification of quantitative and qualitative test methods and reported before [5]. Briefly, Acquity UPLC BEH C18 2.1 × 100 mm, 1.7 μm particles column (Waters, Milford, MA, USA) kept at 40 °C was used for chromatographic separation with a mobile phase composed of water with 0.1% formic acid (A) and acetonitrile with 0.1% formic acid (B). Electrospray ionization (ESI) source was used to introduce the eluent to the mass spectrometer. The appropriate MRM transitions, polarity mode and collision energy (CE) optimized for each compound were used as reported before [5].

### 5.6. MultiMycotoxin Analysis of Urine Samples by HPLC–ESI–HRAMS

An UHPLC Dionex Ultimate 3000 system coupled to Q-Exactive mass spectrometer system was used for quantitative analysis of AFB1, AFB2, AFG2, AFM1, CIT, CPA, FB1, OTA, OTB, ROC, STE, T-2, b-ZEL, a-ZEL in urine samples. Chromatographic peaks were separated on a Waters Acquity UPLC BEH C18 column (2.1 mm × 100 mm, 1.7 μm) at a flow rate of 0.3 mL/min using linear gradient with acetonitrile containing 0.1% formic acid (A) and water containing 0.1% formic acid (B) as follows: 0–1 min, 20% B, 1–8 min, 20–90% B, 8–9 min 90% B, and then the column was re-equilibrated back to 20% B prior to the next injection. The injection volume was 10 μL for analysis. Heated electrospray ionization (HESI) source was used to introduce the LC eluent to the mass spectrometer. The Q-Exactive mass spectrometer operated in a positive mode with spray voltage + 3.5 kV, capillary temperature 300 °C, auxiliary gas heater temperature 250 °C; S-lens RF level 50 V. Full scan, mass range 150–900 m/z, resolution 70,000, automatic gain control (AGC) target 10^6^ and maximum injection time 200 ms. For CID experiments the mass spectrometer was operated in positive mode using NCE 45. Thermo Xcalibur (version 3.0, Thermo Scientific, Bremen, Germany) software was used to control the instrument setup, for acquiring and processing of the data. The method used vas validated according to the National Association of Testing Authorities (NATA) guidelines for the validation and verification of quantitative and qualitative test methods [28]. Method linearity, limit of quantitation (LOQ) and method specific parameters of 14 mycotoxins analysed in urine are given in Table 4. The limit of detection (LOD) for all the values was half the LOQ value.

### 5.7. Serum AOPP Assay

Upon reaction of serum proteins with chlorinated oxidants AOPPs, i.e., uremic toxins, are formed. Serum AOPP were determined according to the method described by Witko-Sarsat and colleagues [29]. The absorbance of the reaction mixture was read at 340 nm on the Tecan M200 Pro plate reader (Tecan, Grödig, Austria) and results expressed as chloramine-T equivalents.

### 5.8. Measurement of TSP

TSP were determined according to the method described by Tatzber and colleagues [30]. The absorbance of the reaction mixture was read at 450 nm on the Tecan M200 Pro plate reader (Tecan, Grödig, Austria) and results expressed as hydrogen peroxide (H_2_O_2_) equivalents.

### 5.9. Thiobarbituric Acid Reactive Substances (TBARS) Assay

The impact of AFB1 on the peroxidation of lipids was measured by the TBARS assay using an 1,1,3,3-Tetraethoxypropane, a MDA precursor, as a standard. To 90 µL of standard or urine samples, 25 µL of water, 25 µL of 42 mM thiobarbituric acid and 75 µL of 1% H_3_PO_4_ was added. After incubation for 30 min at 90 °C, samples were immediately placed on ice to cool down. Samples were then transferred to 96-well black plates and fluorescence measured at excitation 515 nm and emission 556 nm using Tecan M200 PRO (Tecan, Austria).

### 5.10. Urinary DNA/RNA Oxidative Damage Assay

DNA/RNA oxidative damage was measured in urine samples by commercially available enzyme-linked immunosorbent assay (Cayman Chemical, Ann Arbor, MI, USA) following manufacturer protocol. This assay detects 8-hydroxydeoxyguanosine, 8-hydroxyguanosine and 8-hydroxyguanine.

### 5.11. Statistics and Data Analysis

Descriptive statistics were shown as the mean ± standard deviation (SD). The significance of differences between groups was assessed using the Student *t*-test and Chi-square test. When more than two groups were compared, one-way ANOVA was used with appropriate post hoc testing. Statistical calculations were performed with IBM SPSS 25.0 (IBM Corporation, Armonk, NY, USA) and GraphPad PRISM 6.02 (GraphPad Inc., La Jolla, CA, USA) for Microsoft Windows. Differences with hypothesis testing *p*-value less than 0.05 were considered statistically significant.

## Figures and Tables

**Table 1:** Cohort characteristics.

	Urine Samples (*n* = 559)	Serum Samples (*n* = 412)
Female	256	197
Male	303	215
Age (years)	38.5 ± 9.7	39.2 ± 10.1
Weight (kg)	76.8 ± 18.5	76.3 ± 17.3
Overall Health		
Excellent	169	137
Good	289	209
Fair	84	53
Poor	13	8
No answer	4	5
Smoking Status		
Smoker	132	85
Nonsmoker	198	161
Former smoker	42	31
No answer	187	135

**Table 2:** Incidence and concentration range of mycotoxins in serum samples.

Analyte	No of Positive Samples		Total Incidence	Incidence (%)	Range (ng/mL) *
	<LOQ #	>LOQ			
15-AcDON	0	1	1	0.2	8.3
3-AcDON	14	12	26	6.3	3.2–7.2
AFB1	0	0	0	0.0	n.a.
AFB2	0	1	1	0.2	0.2
AFG2	1	25	26	6.3	0.2–12.9
AFM1	0	0	0	0.0	n.a.
Total AFs ^$^	1	26	27	6.6	0.2–12.9
CPA	1	5	6	1.5	0.4–2.6
DON	0	0	0	0.0	n.a.
FusX	0	0	0	0.0	n.a.
HT-2	0	0	0	0.0	n.a.
MPA	40	1	41	10.0	1126.1
NEO	45	0	45	10.9	<LOQ
OTA	15	4	19	4.6	3.9–5.6
OTB	2	1	3	0.7	<LOQ–46
Total OTs	17	5	22	5.0	<LOQ–46
ROC	0	0	0	0.0	n.a.
STE	0	1	1	0.2	0.7
ZEN	0	1	1	0.2	28.1
a-ZEL	0	0	0	0.0	n.a.
b-ZEL	0	0	0	0.0	n.a.

n.a. not applicable.; * The range is given only for positive samples; # Number of positive samples >LOD but <LOQ; ^$^ Total AFs: AFB1 + AFB2 + AFG2 + AFM1.

**Table 3:** Incidence and concentration range of mycotoxins in urine samples.

Analyte	No of Positive Samples		Total Incidence	Incidence (%)	Range (ng/mL) *
	<LOQ #	>LOQ			
AFB1	2	0	2	0.4	<LOQ
AFB2	13	0	13	2.3	<LOQ
AFG2	5	4	9	1.6	0.19–0.34
AFM1	3	0	3	0.5	<LOQ
Total AFs ^$^	23	4	27	4.8	0.19–0.34
CIT	6	0	6	1.1	<LOQ
CPA	0	0	0	0.0	n.a.
FB1	1	0	1	0.2	<LOQ
OTA	0	0	0	0.0	n.a.
OTB	3	0	3	0.5	<LOQ
ROC	18	6	24	4.3	0.21–0.33
STE	5	0	5	0.9	<LOQ
T-2	3	0	3	0.5	<LOQ
b-ZEL	28	14	42	7.5	5.11–19.58
a-ZEL	16	6	22	3.9	5.08–7.01

n.a. not applicable.; * The range is given only for positive samples; # Number of positive samples >LOD but <LOQ.; ^$^ Total AFs: AFB1 + AFB2 + AFG2 + AFM1.

**Table 4:** HPLC–ESI–HRAMS multimycotoxin method linearity (linear range and correlation coefficient (R^2^)), limit of quantification (LOQ) and overview of the compound specific HRMS parameters of 14 mycotoxins measured in human urine.

Analyte	Linear Range (ng/mL)	R^2^	LOQ (ng/mL)	Measured Form/Adduct	Accurate Mass (*m*/*z*)	RT (min)
AFB1	0.2–10.0	>0.994	0.2	[M + H]^+^	313.07066	5.17
AFB2	0.2–8.0	>0.998	0.2	[M + H]^+^	315.08631	4.84
AFG2	0.2–8.0	>0.990	0.2	[M + H]^+^	331.08123	4.45
AFM1	0.2–6.4	>0.985	0.2	[M + H]^+^	329.06558	4.82
CIT	1.5–24.0	>0.990	1.5	[M + H]^+^	251.09140	6.00
CPA	0.2–10.0	>0.994	0.2	[M + H]^+^	337.15467	7.53
FB1	0.2–10.0	>0.995	0.2	[M + H]^+^	722.39575	5.03
OTA	1.5–24.0	>0.989	1.5	[M + H]^+^	404.08954	6.86
OTB	5.0–40.0	>0.990	5.0	[M + H]^+^	370.12851	6.15
ROC	0.2–10.0	>0.992	0.2	[M + H]^+^	390.19245	5.25
STE	0.2–10.0	>0.994	0.2	[M + H]^+^	325.07066	7.08
T-2	1.5–15.0	>0.986	1.5	[M + Na]^+^	489.20950	6.60
a-ZEL	5.0–40.0	>0.994	5.0	[M + H]^+^	321.16965	6.21
b-ZEL	5.0–40.0	>0.991	5.0	[M + H]^+^	321.16965	5.80

## Data Availability

The data that support the findings of this study are available from the corresponding author upon reasonable request.
